# Enabling Older Adults to Provide High-quality Activity Labels: Unpacking Accuracy, Precision, and Granularity in Activity Labeling

**DOI:** 10.1145/3770649

**Published:** 2025

**Authors:** YIWEN WANG, HOSSEIN KHAYAMI, BONGSHIN LEE, AMANDA LAZAR, HERNISA KACORRI, EUN KYOUNG CHOE

**Affiliations:** University of Maryland, College Park, USA; University of Maryland, College Park, USA; Yonsei University, Republic of Korea; University of Maryland, College Park, USA; University of Maryland, College Park, USA; University of Maryland, College Park, USA

**Keywords:** Data labeling, Older adults, Label quality, Activity tracking, Human activity recognition, Co-design, Machine teaching, Personalization

## Abstract

High-quality labels of activity data with broad representations and real-world variability are key to developing activity recognition models tailored to the needs and characteristics of older adults. However, labeling real-world data presents significant challenges, placing a heavy burden on users to provide high-quality labels while staying engaged in their activities. This paper investigates older adults’ perceptions of providing high-quality labels in the context of training their personalized activity trackers. We conducted a co-design study with 12 older adults to envision the labeling process—describing activity names and time spans—using the teachable machines paradigm as a scaffold. We unpack the contextualized definitions of accuracy, precision, and granularity through a thematic analysis of older adults’ perspectives on activity labeling. Our findings present participants’ preferred strategies for obtaining high-quality activity labels with less burden and intrusiveness, including user-initiated labeling and machine-initiated prompting. We discuss design considerations for future data labeling tools that address discrepancies between user perceptions and technical standards in training personalized activity trackers.

## 1 INTRODUCTION

Older adults are underrepresented in training datasets [45, 73], which may contribute to age-related bias in Artificial Intelligence (AI) systems [18]. In general, current technologies for older adults have been developed with a narrow focus, often neglecting individual differences [53]. To accommodate a variety of activities older adults typically perform and perceive as meaningful, ranging from moderate-to-vigorous exercises to hobbies, leisure, and less strenuous activities [56, 67], prior HCI and aging research called for a personalized approach in activity tracking systems [93, 96]. Advancing such personalized activity recognition systems relies on high-quality labels with broad representation and variability, which are essential for developing and fine-tuning the underlying machine learning (ML) model. However, achieving *high-quality labels*—characterized by high accuracy (i.e., accurately timed and correctly named), high precision (i.e., consistently categorizing similar activities under the same conditions), and granular in semantics and contexts as needed—can impose substantial data capture burdens on users. This is particularly challenging when involving end users, many of whom may lack ML familiarity, in interacting with, teaching, and training personalized systems [42, 81].

Wearable and mobile devices equipped with sensing capabilities have shown promise in collecting labeled activity data from participants in their natural environment [15, 51, 54, 91]. To reduce the user burden of remembering labeling tasks and recalling past activities, these labeling tools provide various mechanisms for user input, including selecting multiple labels from a list of pre-defined activity categories and responding to prompts sent via a watch [91]. However, few existing labeling tools offer greater flexibility or are designed with older adults’ preferences and activity scenarios in mind. One exception is the MyMove system [51], which adopted a speech-based approach to engage older adults in activity labeling. While this approach improved accessibility, it also introduced limitations, as nearly half of the reports lacked complete time information, impacting overall data quality. Also, when individuals engage in activities composed of multiple actions (e.g., gardening involves digging, lifting, and moving items), such blurring of activities poses significant challenges for participants to accurately label them and for researchers to effectively analyze them [54, 96]. Thus, a gap remains in understanding how older adults perceive the challenges of activity labeling and how to balance the labeling workload between the user and the system to acquire high-quality labels. Addressing this gap could better align user-generated labels with the technical requirements needed to build effective activity recognition models.

In this paper, we set out to understand how older adults perceive the activity labeling task, with an emphasis on their understanding of what constitutes “high-quality labels” by unpacking the notions of accuracy, precision, and granularity in activity labels. Building on this understanding, we examine participants’ preferred strategies for user-initiated labeling and machine-initiated prompting to collect high-quality labels. This work is informed by prior research on teachable machines, which empower end-users without much ML background to train ML models and create personalized technology that serves their personal goals and needs [26, 38, 43, 70]. End-users could act as “instructors,” actively selecting and labeling activity examples while the machine could learn the concepts conveyed in the teaching examples [81]. Our ultimate goal is to enable older adults in machine training, where the collected high-quality labels can be used to train a personalized activity tracker that caters to their activity traits and tracking needs. While activity labeling may resonate with the concept of self-tracking in many aspects (e.g., collecting information to increase self-knowledge [62]), the labeling process plays a pivotal role in training and developing activity trackers. Yet, we know little about older adults’ preferences and perceptions when teaching a personalized activity tracker via data labeling. Overall, our research questions are: *What are older adults’ perceptions of data accuracy, precision, and granularity in activity labeling? What are the strategies preferred by older adults to provide high-quality labels through interaction with a teachable activity tracker?*

We employ co-design, a participatory method proven effective in involving older adults’ perspectives in the early phase of technology design and development [3, 35, 83, 89]. Our study leverages this approach to design activity labeling tools aimed at empowering older adults to effectively and comfortably train their personalized activity trackers. We conducted a co-design study with 12 older adults (aged 64 to 93) to understand their perceptions of activity labeling and suggestions for labeling mechanisms. Throughout a qualitative data analysis, we first unpacked the meaning of accuracy, precision, and granularity in labeling activity names and time spans for participants, noting how these aspects are influenced by participants’ contextual differences and their activity goals. Participants also provided suggestions for user-initiated labeling and machine-initiated prompting to support them in providing high-quality activity labels. Specifically, participants opted to adjust the granularity of labels at different stages and control the prompt timing to reduce the high burden of labeling in situ. Meanwhile, machines can ensure and verify label completeness, encourage reflection on activity boundaries, and elicit finer details through prompting. Our findings suggest the design implications that align the user perception and machine training requirements in collecting high-quality activity labels. Our contributions are as follows:

- We report on participants’ perception of collecting high-quality labels by examining their understanding of accuracy, precision, and granularity in the context of activity labeling, along with our contextualized definitions of these terms.
- We present insights on the user-initiated strategies and machine-initiated prompting by foregrounding participants’ perception of activity labeling in the context of activity name and time span.
- We discuss design considerations on a mixed-initiated approach that addresses the gap between user-generated labels and the technical requirements in training personalized activity trackers.

## 2 RELATED WORK

This section first reviews common self-reporting strategies to capture activity information: experience sampling and retrospective recalling. We then present the user experience of these labeling systems and discuss the challenge of misalignment between users and the system. Further, we review the definition of accuracy in activity tracking systems, including those adopted by older adults.

### 2.1 Self-reporting Approaches to Understand Daily Activity

Substantial research has focused on self-reporting approaches for capturing daily activity, either in situ or through retrospective recall. The Experience Sampling Method (ESM), also known as Ecological Momentary Assessment (EMA) [55], prompts participants to report their activity experiences multiple times a day [92], often leveraging wearable or mobile devices to deliver timely and minimally disruptive notifications [36]. Compared to traditional diary studies that fully rely on participant-initiated entries [8], ESM reduces recall bias and improves data quality [92] with prompts ranging from open-ended to binary and multiple-choice questions [1, 57, 77].

In addition to ESM, the Day Reconstruction Method (DRM) captures activity experience, context, and time use by dividing the day into episodes via survey questions [44]. This method reduces the data capture burden, but the recall bias remains, leading to potentially inaccurate reports [23]. Individual differences—such as memory-experience gaps [28] and cognitive demand of different activities [97, 98]—can also influence the reporting experience and burden.

Based on the pros and cons of these self-reporting strategies, there is an ongoing debate about whether experience sampling or retrospective recall yields more accurate and precise outcomes [28, 64, 84]. A key distinction lies in capturing subjective experiences (e.g., feeling) versus the objective reality of timestamped activities. Here, there is a prominent gap in understanding how those self-reporting strategies can complement each other, combining voluntary reporting, prompting mechanisms, and retrospective recalling to enhance the data quality. Informed by prior work, this paper aims to investigate user-initiated labeling and machine-initiated prompting strategies that facilitate reporting activity names and time spans in situ or recall afterward.

### 2.2 Current Activity Labeling Systems and Tradeoffs

Personalizing or fine-tuning human activity recognition (HAR) models typically involves activity labeling. To collect activity labels in situ, prior work has adopted a wide range of interaction modalities, ranging from touch input for selecting from a list [54, 91], voice input [1] to multimodal interaction that involves multiple sensors, devices, or modalities [22, 90]. These modalities are varied in pros and cons regarding the user burden and data quality. For example, adopting the *μ*EMA technique, Ponnada et al. explored self-reporting on a smartwatch that allows users to select activity intensity with a simple tap [77]. While effective in reducing the data capture burden, participants occasionally missed prompts during vigorous activities. Additionally, voice input enables hands-free, in-situ reporting on wearable devices (e.g., a wrist-worn [1] and an earable-based system [57]). However, participants reported discomfort with responding aloud in public and wearing devices, and concerns about the audio quality. Multimodal systems have further expanded the annotation toolkit. Tonkin et al. [90] incorporated voice, NFC tags, selection from lists, and manual entry. Other systems use multiple sensors and devices—such as smartwatches, smart glasses, and beacons—to support labeling complex activities like eating, cooking, and grabbing [22, 24]. However, these approaches have predominantly been tested with younger users, leaving older adults’ perspectives underexplored.

Of current activity labeling systems, increasing flexibility often leads to compromised data quality, depending on how the system classifies activities. The Extrasensory app lets users select from pre-defined categories, including body posture (e.g., “sitting”, “walking”) or contextual information (e.g., “eating at school”) [91]. However, this list of activities is not customizable, and collecting labels following the pre-defined categories places a significant burden on users, affecting the authenticity of their behaviors. Another labeling system allows users to customize labels for hand activities—a series of hand actions, such as “clapping” or “typing” [54]. However, researchers experienced difficulties in understanding the customized categories created by participants [54, 90]. The reported labels varied in granularity—some referred to compound activities like “eating” and “cooking” that contain a series of components like washing, chopping, and mixing, while others are more fundamental and atomic. Additionally, some labels are ambiguous, making it challenging for the researchers to gauge the precise meaning. As such, participants’ perception of labeling doesn’t always translate to researchers. Participants believed that their labels were helpful and invested effort into capturing them. However, labels collected with greater flexibility often lacked the quality needed to effectively train ML models. We suspect that users’ understanding of what constitutes “high-quality labels” (i.e., users’ conceptual model) did not align with the requirements needed to build effective activity recognition models (i.e., the system model of how it actually functions).

To lower the labeling burden in real-world scenarios, researchers have begun exploring labeling systems designed for older adults [25, 51]. The MyMove system—a speech-based labeling approach on a smartwatch, incorporating both voluntary reporting and a prompting mechanism (i.e., ESM)—was developed to offer greater flexibility in labeling for older adults [51]. While participants were highly engaged in the labeling process via verbal reporting, the lack of complete activity time information—specifically both start and end times—diminished the overall usefulness of the labels. Additionally, complemented by a traditional diary-based method, older participants could create more labels by reviewing the entries retrospectively [25]. To maintain data quality, prior work proposed a semi-automated approach—combining user input with algorithmic support—in systems using sound detection [21] or prompting participants to label by identifying transitions between movements (e.g., walking to standing) [2, 19]. While automated labeling or prompting to label can be convenient, users sometimes prefer manual input for greater control [15]. Still, older adults’ preferred strategies and perception of collecting high-quality labels remain underexplored. Informed by the prior work, we aim to investigate older adults’ preferred strategies on user-initiated labeling and machine-initiated prompting, and how to ensure the data quality while reducing the user burden.

### 2.3 Accuracy in Activity Tracking and High-quality Labels for Older Adults

Wearable activity trackers (WAT) quantify one’s physical activities via various metrics (e.g., step counts, distance, heart rate). However, concerns about the accuracy of sensor-recorded data have increasingly been raised in the context of older adults’ usage [49, 80, 82]. For example, the tracker incorrectly counted the wake-up times [30, 80], steps [11], and stride distance [29]. This tracking accuracy issue could potentially influence the device’s adoption by older adults. A substantial study has examined the accuracy and precision of WAT with diverse user groups, including younger adults [27] and older adults [75], children and adolescents [66], adults with Parkinson’s disease [14], and people with multiple sclerosis [72]. Most of these WAT studies focused on evaluating the accuracy and reliability of collecting data, such as step counts, by comparing multiple devices. However, the user-perceived definition of accuracy is left out [86]. Among the objective measurements, *accuracy* refers to the closeness of measured values to the gold-standard measuring device, where the measuring device is understood as having captured the truth [4, 27, 75, 95]. The definition and interpretation of *precision* vary depending on the research context. In many scientific fields (e.g., experimental science), precision refers to the repeatability or consistency of measurements when the same device or method is used to perform the same task multiple times under identical conditions [66]. In ML, however, precision is defined as the proportion of true positives (i.e., correctly predicted positive cases) out of all predicted positives (i.e., true positives + false positives) [20]. In the context of data labeling, the former definition—repeatability or consistency—is more relevant, as the user acts as a data labeler (or ‘measurement’), and maintaining a consistent labeling practice is critical for ensuring label quality.

In contrast to the established definitions of accuracy and precision in scientific literature, we know little about older adults’ perception of these concepts and how their understanding influences the quality of labels they provide for training HAR models that, in turn, benefit end-users. Considering the wide range of activities older adults partake in, current HAR models might not be accurate or precise when used by people with diverse backgrounds. Specifically, prior work observed a declined accuracy when detecting activities with slow walking speed [11, 72] or movements with short duration (e.g., bending or crouching) [41]. Due to a lack of reliable measurement of low-exertion activities, older adults may overexert themselves by completing activities that exceed their physical abilities [95]. The data accuracy issues might be elevated by diminished label quality, such as unreliable ground-truth data [40, 72]. Also, people may not be likely to carry their devices when performing daily activities of living like cleaning and cooking [75], leading to a scarcity of such activities. Our work aims to bridge the gap in understanding older adults’ perception of data accuracy and precision with the goal of providing high-quality labels to train a personalized activity tracker.

In our paper, we build upon prior work on WAT systems for older adults [93], which has primarily focused on a limited set of typical activities such as walking and exercising. Drawing from existing activity labeling systems [25, 51, 91], we conduct a co-design study by involving older adults as collaborating partners in the early phase of system design. We analyze participants’ perceptions of activity labeling and their preferred strategies for collecting activity labels in situ. Specifically, we examine the concept of “high-quality labels” based on our contextualized definitions of accuracy, precision, and granularity, as shown in Table 2.

## 3 METHOD

**Fig. 1.**
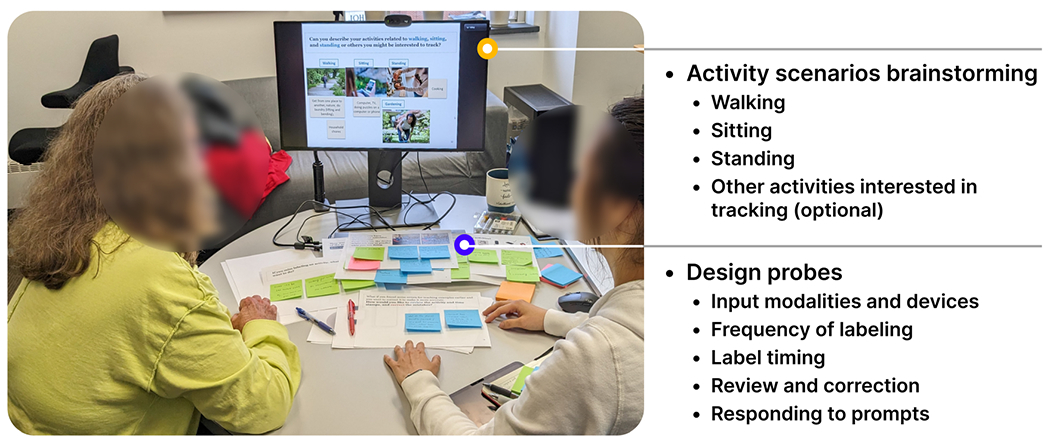
The design activities consist of (1) activity scenarios brainstorming: we asked participants to describe their typical activity scenarios, including walking, sitting, standing, and any activities they are interested in tracking; (2) design probes: we situated participants in their relevant activity scenarios and used design probes in a semi-structured interview format.

We conducted a co-design study (Figure 1) with 12 participants either in person in a quiet lab setting or remotely via Zoom. Through the co-design activities, participants shared their understanding and preferred strategies for capturing high-quality activity labels through interacting with a teachable activity tracker. This work takes place within a larger project aimed at collecting diverse labeled activity data that reflect older adults’ activity patterns for developing personalized technologies that promote healthy and active lifestyles in the long run. This study was approved by the lead author’s University Institutional Review Board.

### 3.1 Study Design and Procedure

This co-design study aims to elicit participants’ preferences, concerns, and design insights about two labeling mechanisms—user-initiated labeling and machine-initiated prompting—to create high-quality activity labels for machine training purposes. We adopted a scenario-based design method [13] to create user-centered solutions with participants who have lived experience and diverse technology backgrounds. Scenarios are the context of human activities. They set the constraints when users interact with technologies—in our case, activity labeling systems. We provided a concept tutorial and design worksheet, created by four researchers with expertise in HCI, accessibility, and ML, to serve as ramps for translating technical terms (e.g., data labeling, teachable machine, training process) into layman’s language. We piloted the study procedure with two older adults who are retired HCI and Accessibility researchers to develop a shared language between the senior community and the researchers and developers. The study consisted of the following four parts.

#### 3.1.1 Part 1: Study Introduction.

We began the study with a brief self-introduction regarding participants’ retirement status, last employment, and routine activities. We then introduced the study motivation and goal, which is to develop a personalized activity tracker. We emphasized that the concept of activities should not be limited to traditional physical activities and exercises but should encompass a wide range of activities that individuals find important to track. In addition, we highlighted concerns about the accuracy of the device and the lack of efforts to involve older adults in the technology design and development process.

#### 3.1.2 Part 2: Tutorial and Warm-up Activity.

**Fig. 2.**
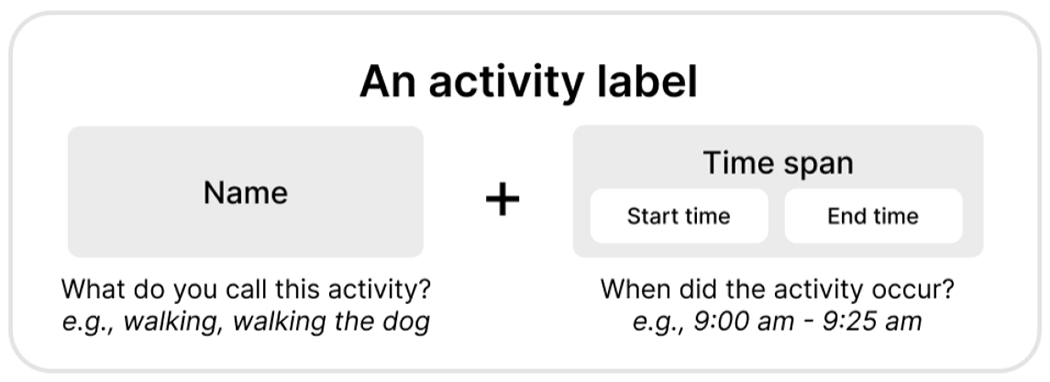
Labels refer to a descriptive tag assigned to a specific activity. Each label should have both activity name and activity time span (i.e., start time and end time).

Inspired by prior work on machine teaching, the tutorial started with a question about participants’ previous experiences teaching someone how to perform activities [26]. We provided examples of how an instructor would demonstrate different postures for stretching or various chords and hand movements for playing the guitar. Through this prompting question, we introduced one important teaching principle—providing good and diverse examples is important when teaching a machine. To further illustrate the teachable machine concept, we presented a video of the Google Teachable Machine 2.0 [32], which demonstrates how machines can be taught to suit individual needs through inputs and outputs. Then, we presented wearable devices equipped with multiple sensors and explained the concept of activity labels. Specifically, labels refer to *a descriptive tag assigned to a specific activity*, including both the activity’s *name* and its *time span*, as shown in Figure 2. We demonstrated a teaching pipeline consisting of data labeling, training, and testing.

Moreover, we introduced various labeling methods, including traditional paper-based diaries, digital calendars, and more advanced solutions on smartwatches, smart speakers, and mobile phones. We also explained input modalities, such as voice input, selecting from a list, text entry, gestures, and the prompting feature. As a warm-up activity, participants were asked to label two activity contexts—(1) walking around the house, (2) slow walking and then sitting down—by providing both the activity name and time span. We highlighted that there are no right or wrong answers due to the subjectivity of labeling.

#### 3.1.3 Part 3: Design Activities.

**Fig. 3.**
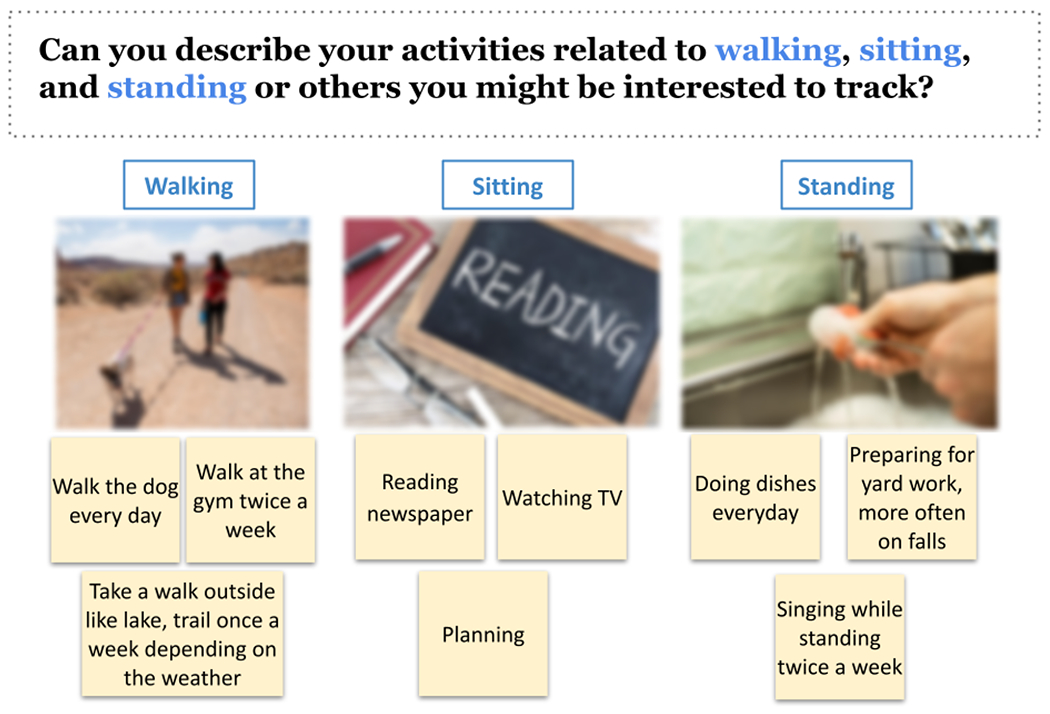
Example of activity scenarios created by participant P4 in Google Slides. Using the built-in image search, P4 selected images representing herself performing walking, sitting, and standing activities.

We adopted a scenario-based design approach [13] to explore how older adults interact with activity labeling systems in their daily living contexts [78], as an example shown in Figure 3. Participants first described their everyday activity scenarios centered around sitting, standing, walking, and other types. To make the design session more relatable, they were encouraged to add images that represent their scenarios using the image search within Google Slides [33]. Following that, we provided design probes to elicit participants’ feedback regarding user-initiated labeling and machine-initiated prompting strategies in a semi-structured format Figure 1. Topics include (1)input modalities (e.g., voice input, selecting from a list, text entry, gestures, and responding to yes/no questions) and devices (e.g., smartwatches, mobile phones, and smart speakers), (2) frequency of labeling, (3) label timing (i.e., before, during, right after, or sometime later), (4) reviewing and correction, (5) responding to prompts (e.g., the device might predict what you are doing). During the co-design process, one facilitator captured key ideas on sticky notes as the conversation went on. We shared the notes with participants in real time to eliminate the writing efforts and enhance the study engagement [9].

### 3.2 Participants

We recruited 12 participants aged 64 to 93 (5 Male and 7 Female) with diverse backgrounds and self-rated confidence in technology use, shown in Table 1. We considered the gender balance when recruiting. We advertised through senior community mailing lists in the Northeast and Northwest regions of the U.S. Our inclusion criteria required participants who were aged 60 or older and to have a minimum interest in enhancing awareness of everyday activities and interacting with technology in general. This requirement ensures participants provide design insights that fit into their real-world needs. All participants had experience interacting with smartwatches, and 9 were returning participants in our lab. 11 out of 12 were smartphone users. 5 participants had actively used Amazon Echo Dot (Alexa). Half of the participants attended the in-person session, while the other half chose the online version. Our sample leans heavily toward a highly educated population. 11 out of 12 participants held at least a bachelor’s degree or above. Participants noted various health conditions during the study, including pre-diabetes (P1), diabetes (P2, P12), high blood pressure (P3), high cholesterol (P7), HIV, osteoarthritis, a history of cancer, depression, anxiety, and degenerative disk disease (P11). P5 had a recent hip replacement surgery, and P6 was taking medication. One participant noted having difficulty hearing and using hearing aids. All participants were compensated by $50 for completing a study lasting 2 to 2.5 hours (approximately $20 per hour).

### 3.3 Data Analysis

We video recorded the study, transcribed the audio, and captured images of the design worksheets. We conducted a codebook thematic analysis using both inductive and deductive coding approaches [10]. In the first round of coding, the lead author familiarized herself with the transcriptions, along with the memo noting her point of interest. The initial coding framework is developed based on concepts such as user-initiated labeling and machine-initiated prompting. The lead author coded P1–P4, while refining and expanding the coding framework along the way. The updated codebook included codes related to participants’ perceptions of the labeling process and the activity labels, preferred strategies, challenges, concerns, and how machines can assist them in the activity labeling. The lead author and senior author held weekly meetings to discuss the coded data and the evolving coding framework. During these discussions, they identified emerging themes regarding how users’ labeling practices aligned or did not align with the goal of providing high-quality activity labels. As such, we developed the contextual definitions of accuracy, precision, and granularity (i.e., how these terms are defined in the context of activity labeling, as shown in Table 2). These definitions served as a guiding framework for re-analyzing participants’ perceptions of activity labeling—through the lenses of accuracy, precision, and granularity. In the second round of coding, one researcher re-coded the data. Similar to the first round, the lead author and senior author met weekly to discuss the coding framework, clarify confusing concepts, and finalize the themes, which included: user-initiated labeling and machine-initiated prompting, both in the context of *activity name* and *activity time span*.

## 4 FINDINGS

In this section, we present participants’ understanding of high-quality labeling based on their perception of accuracy, precision, and granularity. We draw the definitions of those key terms from existing notions. ***Accuracy*** is defined as the closeness of a measured value to the true value [68]. ***Precision*** refers to the closeness of agreement between repeated measurements carried out multiple times in the same condition [68]. ***Granularity*** refers to the level of detail or depth of the data [48]. We contextualized these definitions within activity labeling, as summarized in Table 3, and used them as a guiding framework to examine participants’ perceptions of the labeling process and preferred strategies (in bold) for achieving high-quality labels.

### 4.1 Perceived High-quality in Activity Name and Preferred Labeling Strategies

#### 4.1.1 Accuracy in Activity Name and Contextual Differences.

In the context of activity labeling, *accuracy* of an activity name would be correctly identifying and labeling an activity as it truly occurs. For instance, if someone is doing a mixed-up yoga pose, labeling it as “yoga” rather than “sitting” would be accurate. However, a key challenge in activity labeling is the absence of an absolute or universally agreed-upon “ground truth” label, making the task inherently subjective. Ambiguous activities may have multiple valid labels—for instance, “yoga” could also be labeled as “balance training” or “stretching.” Those alternative ones are reasonably accurate. Interpretations can vary, meaning there might not be a single, definitive ground truth. Participants’ perceptions of the accuracy of an activity name varied based on their activity context and individual views of their performance. During the warm-up activity, participants labeled a hypothetical scenario where they had just finished a slow walk for 35 minutes. The label “slow walk” was noted as not being able to accurately describe participants’ unique patterns of walking, considering different contexts related to walking. For example, P4 criticized the activity name “slow walk” for “*not being specific enough*” to represent scenarios like walking with a dog, during a gym class, or with a partner. Moreover, the term “slow” is subjective in nature, meaning it is hard to assess one’s speed without a concrete reference point. P8 explained, “*Sometimes I think I’m going faster than I was, but a lot of times I’m not*.” These examples illustrate the inherent challenges in achieving universally accurate activity labels, as individual perceptions and contextual differences could influence how an activity is described.

##### Ensuring label completeness through machine-initiated suggestions.

Although a universally agreed-upon accurate and correct label is hard to achieve, participants strive to ensure the completeness of a dataset through a machine-prompting feature to suggest an activity that truly occurs. Completeness can apply to an individual label, ensuring that all relevant aspects of an activity are captured and labeled or to the dataset as a whole, meaning it includes a wide range of (or a selected few) activities necessary for a comprehensive understanding of the phenomena being studied. P5 envisioned the machine prompting her to capture labels based on its knowledge of her past activities, including regular shopping on Wednesday and physical therapy on Tuesday and Thursday: “*If it is like they’ve been using it for a while, and it kind of knows what my activities are there, it has a feeling for my activities. If the machine senses that, it knows. And it could predict what I’m gonna do from one day to the next*.” Additionally, the machine could recognize fluctuations in routine activities and prompt users when a label might be missing. As P1 suggested, “*At the end of a day, if there’s no dog walking activity or anything that I do on a consistent basis, it would be nice if it would ask me, ‘Did you walk the dog today?’ or something to recognize that you do this, five days a week, but on this particular day, I don’t see this activity*.” However, participants acknowledged that predicting future activities accurately daily may be challenging, as people often adapt to changes in their schedules. For instance, P6 mentioned how she occasionally replaces her usual Saturday rapid walk or light run with a solitary walk on a trail to achieve a similar activity level.

#### 4.1.2 Precision in Activity Name and Personal Significance.

In the context of activity labeling, we define *precision* of an activity name as categorizing and labeling similar activities consistently based on specific conditions, even if those labels might not always be accurate. For example, if “running” is the label used for a specific type of activity, precision means always using “running” and not sometimes using “jogging” or “sprinting” for similar activities. Therefore, to collect highly precise labeled data, one must consistently apply the same label to similar activities and clearly delineate boundaries between distinct activities when necessary. Participants distinguished between similar activities based on context, such as who they are with or where the activity takes place. For example, P2 differentiates between biking alone, associated with higher exertion, and slow biking with a partner. Similarly, P6 considered strolling—walking at a slower pace—referring to the case when she walks the dog, which is different from her typical walking. Participants’ decisions to distinguish between activities were also influenced by their goals and the personal significance of each activity. For example, P10 and P11 desire to do more dedicated and active walking, intentionally choosing not to label indoor ambulation that is not worth tracking. Among the two types of walking P10 identified (i.e., walking for commuting and exercise versus walking the dog and moving around the house), he desires to teach only the first kind of active walking that is over five minutes. Likewise, although the exertion level of indoor and outdoor walking could be roughly the same, P11 conducted those activities to fulfill different goals. Specifically, dedicated walking is for “getting out of the house.” As such, activity labeling is not just a matter of accurately identifying what someone is doing; it also involves understanding the underlying intentions, goals, and contextual nuances that make one activity distinct from another, even if they appear similar on the surface.

##### Identifying and prompting for new activities beyond existing labels.

As participants suggested, machines could give feedback when users engage in a new activity for the purpose of assigning labels precisely across a variety of activity contexts. The machines could prompt users to draw a line between existing and new activities, enabling more accurate and precise labeling. For example, P2 suggested that the machine could notify users of new activities and seek a human response on its label, “‘*Well, looks like you were doing something different here, here and here. We will have the labels for that. Any idea what that was?*’”. However, P2 also noted that his activity routine is settled, so he would rarely need to add new activity labels. Similarly, P5’s comment also echoes the idea of sensing differences between known activities and prompting her for new labels. What’s more, P8 suggested machines’ prompting feature to assist her while going through a menu-like list of existing categories and selecting an appropriate one. She envisions receiving prompts when she inputs a new label, saying that “‘*Okay, we are adding an, we are identifying a new activity*.’”

#### 4.1.3 Granularity in Activity Name and Labeling Burden and Benefits.

In labeling activity names, we define *granularity* as the level of detail in the labels, whether related to the semantics of the activity or its contextual specifics. Granularity and accuracy are related but distinct concepts in activity labeling; accuracy is about correctness, while granularity is about the depth or specificity of the label. For example, a fine-grained label for a yoga activity might specify that exact pose (e.g., downward-facing dog), whereas a coarse-grained label might simply be a broader category like “yoga”. Providing highly detailed, fine-grained labels can increase the labeling burden, whereas coarse-grained labels reduce this burden, as P8 explained: “*The more refined you are in categories, the harder it is to draw a line between them*.” Hence, it is important to strike a balance between the level of granularity and the associated labeling burden.

Participants recognized that creating fine-grained labels requires more effort and were interested in knowing the benefits they would gain from providing such labels. For example, when fine-grained labeling is necessary, P8 wondered whether machine training could provide her with a tool to quantify the “*physical rewards*” of activities important to seniors. She wishes to gain self-knowledge by answering a list of the following questions associated with her activity goals: “*Am I doing enough of the resistance and weight bearing? Am I doing enough cardio? Am I doing enough—balance is another thing. Do seniors need a lot of work? Am I doing enough for that?… But mostly, I want to be able to measure: Am I doing what I need to do to attain my goals?*”

##### Adjusting label granularity to manage the labeling burden.

A recurring idea for managing the labeling burden is to adjust the level of granularity iteratively across different stages of the labeling process, ranging from the initial labeling for training the basic model to iterated labeling for fine-tuning the model after training. P2, for example, initially provided contextual, fine-grained labels like “*sitting and eating, (or) sitting and eating and reading*” to explore potential physiological patterns. However, he would increase or reduce the granularity if the changes (e.g., body temperature) detected by the activity tracker have or have no meaningful significance.

Participants also discussed adjusting granularity based on the timing of the activity—whether it is before, during, or after the activity is performed. During the activity, when they might be actively engaged, both P1 and P8 preferred initially applying a coarse-grained label, such as “walking,” and then refining it later (e.g., at the end of the activity or later in the day) with more specific subcategories like “*indoor walking or level walking or hill walking*.” Additionally, P1 suggested using a placeholder term to quickly capture unstructured, spontaneous activities, which could then be refined later.

“My [placeholder] word is piddling. But I’m thinking if there’s something that would timestamp for whoever the user is at that time, and then they could come back and fill in what that activity is (…) For me, that [piddling] would be a quick common word that I can use to identify the fact that I’m doing something around the house.”

##### Probing fine-grained contextual information from users.

Participants envisioned a collaborative relationship between users and teachable machines, where machine-initiated prompts could refine the granularity of activity labels. Machines could initiate prompts with coarse-grained labels to probe more details, while users could provide responses that are rich in semantics or contexts. Participants imagined receiving prompts aiming for different granularity. For example, coarse-grained prompts could focus on body postures, such as walking, standing, or sitting, whereas fine-grained prompts would capture more nuanced physical activities or specific types of exercise.

Moreover, researchers, as co-designers, introduced the idea of machines prompting users to confirm ongoing activities. P11, for example, suggested that machines could ask for more contextual information around an activity, such as “*Are you sitting? Are you sitting around eating dinner? Are you sitting at a meeting?*” to better understand the context in which activities occur. However, many participants vacillated on the intrusiveness of machine-initiated prompts. Some found it annoying unless directly related to health-related purposes, like taking medicine (P3), while others were concerned that privacy and agency would be violated. P12 explained that a lack of transparency in how algorithms of machines operate could deter him from engaging with prompts. He expressed concern about anthropomorphism in the prompting feature: “*Prompts are frightening because it’s all of a sudden, all of a sudden I hear it say ‘Are you exercising today?’ Well, if it was a human being, I wouldn’t mind it. But it’s some artificial intelligence. I don’t know who’s monitoring this, either*.” To mitigate these concerns, customizable features, such as the ability to adjust the timing of prompts (P12) or opt for reminders to be sent later (P2, P4, P6, P7, P8) are preferable by participants.

### 4.2 Perceived High-quality in Activity Time Span and Preferred Labeling Strategies

#### 4.2.1 Accuracy in Activity Time Span and Demarcation Challenges.

Similar to the concept of accuracy in activity name, accuracy in an activity’s time span means capturing both start and end times that closely align with the actual moments the activity began and ended. Alternatively, capturing either the start or end time along with the duration allows for calculating the complete time span.

Participants agreed upon the labeling requirement of capturing accurate and complete time spans but raised concerns over the difficulty of demarcation—marking the boundary between activities physically by identifying the ground-truth start and end times. For example, P5 described a sequence of activities around cooking, including standing up, walking to the kitchen, washing dishes, preparing and eating lunch, then cleaning up. She emphasized the need for immediate data capture given the difficulty of remembering the accurate start/end times for each component and explained that “*Because if there’s a sequence of events, they all get mushed together. And I don’t have to guesstimate, and I don’t like guesstimating because I’m usually off, and you want accurate time*.” Additionally, demarcating activity boundaries requires significant cognitive effort, which could disengage participants from their main task and potentially pose safety risks. P11, a caregiver in rehabilitation, emphasized the challenges of maintaining proper posture while simultaneously labeling the activity.

##### Supporting immediacy and proximity while alleviating prompting intrusiveness.

To enhance the label accuracy by minimizing the gap between labeling and the actual activity, using natural user interfaces like voice commands and proximity devices such as wearables can be effective. For example, participants imagined wearable devices like smartwatches could eliminate the need to fetch devices and the interruption in the middle of the activity (P6), which is especially convenient for those who do not typically carry mobile phones (P3, P6, P10). P5 preferred verbally interacting with a smart speaker, noting that it helps with chronological data capture and marking the transition between activities: “*tell her (Alexa) what I’m doing, and when I finish, and then what my next move is, then just let her keep the record. And I think it would be more accurate in terms of the beginning and the end times of the activity*.”

Participants also suggested that prompting can enhance both the planning and recording of activities. P12 proposed that the machine can provide an overview of scheduled activities at the beginning of the day, helping users structure their tasks while generating draft labels with activity names and time spans to be reviewed later: “it spits out a calendar to me in the morning…*You know, I turn it on in the morning, and it already knows some of my activities, I’m assuming. And it just sort of goes through a list and says: remember to exercise if you need to go shopping, do your laundry, pay your bills*.” This type of prompting could be helpful when a user’s schedule is more routine. However, P6 preferred not to receive prompts too early, stating, “*I don’t think it’s necessary. I haven’t really done anything*.” This highlights the need for prompts to be well-timed, reinforcing the importance of customization to ensure they are useful and not intrusive. Participants desired to customize prompt timing to fit their schedules. For P6, “*one o’clock midday would be the safest, least intrusive time. I would either be preparing to have lunch, just finishing lunch, [or] thinking about my next transition*.”

##### Improving label completeness through prompting half-baked labeling.

Machine-initiated prompting can assist in improving the completeness of time span data in activity labels. Participants expressed that it would be difficult to provide an accurate duration or end time for activities by predicting before the activity, which might result in incomplete time span records with only the start time captured. They preferred to leave the end time open to avoid inaccurate guesstimation (P4, P6, P7, P11) and to fill it in afterward. For example, P7 valued the flexibility to adjust the duration of activities, such as walking the dog, based on whether she wanted to shorten or extend the time. P2, who was attentive at remembering the start times, imagined that a machine prompting him to log the end time would be helpful, like how his Strava app prompts him to mark the end time of a bike ride: “*It [Strava] reminds me to shut it off, I don’t usually go too long after a ride without shutting it off. And it’s only because it’s telling me. It’s not because I remember, but I do, I’m pretty good at remembering the start*.”

#### 4.2.2 Precision in Activity Time Span and Tolerance for Deviations.

In the context of activity time span, *precision* refers to consistently recording time under the same conditions in which the activity occurs, even if there is a slight deviation from the exact start and end times. Our analysis revealed that tolerance for deviations varies among individuals based on their expectations for labeling accuracy. For instance, P6 noted, “*A matter of minutes, or a matter of half an hour, or a percent of 10% dissolve for something isn’t a terrible thing*.” P5 accepted minute-level deviations but was concerned about hour-level discrepancies, as these were outside the typical range of her walk routine. Similarly, P4, who owns an elderly dog, found that the usual dog-walking duration falls within a 20 to 30-minute range, indicating a practical threshold for acceptable precision. Thus, typical activity patterns can play a role in shaping tolerance levels for deviations.

##### Revealing the level of alignment between time span and machine-sensed data.

Participants evaluated how well machine-recorded times aligned with their subjective reports. For example, P1 questioned the reliability of labeling when discrepancies arose between the duration recorded and the start time, asking, “*I knew I walked around the house for 15 minutes, sometime in the morning. Do I throw off this process by recording that 15 minutes from 10 to 10:15 when it was actually the devices has stored that I did that from 9:45 to 10?*” While the duration is accurate, ensuring close alignment between recorded times and actual activity times is also critical regarding label quality. However, what appears objective, such as time span, may become less so when capturing activities, as ground truth often depends on individuals’ own perceptions of their activities. Therefore, examining the alignment between participants’ reported activity time span and machine-sensed data could help bridge the gap between the mental model of those providing labels and the machine, ultimately minimizing significant errors.

#### 4.2.3 Granularity in Activity Time Span and Recalling Efforts.

In labeling activity time span, we define *Granularity* as the finest unit of time captured (e.g., second, minute, hour). A coarse-grained time span (i.e., recording the time at the higher unit, like hours) could be associated with a generalized activity name, while a fine-grained time span (i.e., recording the lower unit of time, like minutes or seconds) corresponds with a more specific activity description in terms of semantics or context. Participants mentioned the challenge of recalling the activity time span for labeling activities when they were preoccupied. P2 stated the recalling challenge as the memory fades away across time “*as to the times along the way that it’s going to be a little bit of estimating. If I’m not doing it as I go, I can easily envision myself forgetting to actually record what’s happening if something comes up*.”

##### Recalling activity time span from names at different levels of granularity.

In some cases where participants forgot to label the activity in real-time, they chose to capture it afterward by reconstructing the day and recalling the activity context at varying levels of detail. For example, P1 described repopulating the time slot by retrospectively reviewing daily activities in chronological order, using coarse-grained labels (e.g., standing, sitting) to approximate time intervals. “*I’m not concerned that [what] it was at that point, I’m not concerned that it was dog walking versus vacuuming. But I was standing from nine to whatever activities I was doing, it was standing from nine to 11, it was sitting from 12 to 2, something like that*.” Similarly, P6 reconstructed the time by transitioning from the coarse to fine-grained label at the same hierarchy “*Yeah, it’s gonna be hard to have time when it’s casual walking. Only, but it isn’t hard to differentiate it from sitting. So from eight to 12, how much of the time was spent walking? Cleaning… How much time was spent sitting? Reading the paper, doing Sudoku*.” What’s more, P4 estimated the events by reconstructing the day and recalling contextual details at a fine-grained level, like “watching TV” and context around the activity, such as a companion “with husband” and the environmental factor “on a rainy day.”

## 5 DISCUSSION

In this paper, we introduced the contextualized definitions of accuracy, precision, and granularity (Table 2) and examined participants’ labeling challenges and preferences for a mixed-initiated method [39] that blends user-initiated labeling with machine-initiated prompting (summarized in Table 3). While prior work has focused on activity labeling tools for older adults based on ESM [25, 51], we extend this research by investigating older adults’ perspectives on how to collect high-quality labels and mitigate the labeling burden in training personalized activity trackers. It is, however, important to note that older adults are not a homogeneous group, and our participants skewed toward more tech-savvy, highly educated individuals. Therefore, our findings should be interpreted within this specific context, and some insights may not be unique to older adults.

### 5.1 Supporting Low-burden, High-quality Activity Labeling

#### Correctly named.

Our findings suggest that labeling activities in a free-living environment presents substantial challenges, particularly in the absence of a predefined list of activities. This difficulty arises because contextual interpretations of activities are often subjective. Some prior HAR studies with older adult participants typically involve scripted tasks or labeling from a predefined list [12, 41, 100]. Extending on prior work, we observed that participants assess the correctness of an activity label in diverse ways. A label may be perceived as incorrect either because it doesn’t reflect personally relevant scenarios (e.g., P4’s varied walking scenarios) or because the participant struggles to assess the objective ground truth (e.g., P8’s uncertainty about his own perception of walking speed). To support users to accurately label an activity name, future design could provide objective cues derived from sensor data. For example, Khayami et al. adopted the consensus approach [50]—triangulating sensor data, video recordings, and subjective verbal reports—to obtain ground-truth activity labels in older adults. This approach could be integrated into activity labeling systems to help users reconcile subjective perceptions with objective measurements.

Correctly naming an activity can also be challenging due to ambiguity, transitions, or overlapping behaviors. Soft labeling addresses this by assigning probabilistic scores to multiple activity classes—for example, [Walking: 0.7, Running: 0.2, Other: 0.1]—to reflect model uncertainty and avoid overconfidence. These labels can be generated by the system based on confidence scores or, in future user-in-the-loop designs, provided by users to express uncertainty. This approach aligns with active learning, where the system prompts users only when confidence is low. Soft labeling may be more helpful for older adults experiencing cognitive challenges, as it reduces cognitive load to choose a single label and supports more intuitive, user-driven labeling, an idea echoed by participants in our study who advocated for machine-initiated prompting when needed.

#### Accurately timed.

Another challenge lies in accurately marking the start and end times of activities to capture complete ground-truth labels, a difficulty also highlighted in prior work on speech-based labeling with older adults [51]. This task requires significant cognitive efforts, particularly for complex, multi-step activities such as cooking (P5), which often involve composite actions like sitting, standing, walking, washing dishes, etc. Participants emphasized the need for immediacy and proximity when capturing sequences of activities to ensure the quality of labeling while minimizing disruption. To balance label completeness, accuracy, and user burden, we envision a multimodal approach that prompts users at different times. Such a system could combine: (1) *pre-labeling*: allowing users to generate a temporary label by planning upcoming activities; (2) *real-time marking*: enabling in-the-moment labeling through *μ*EMA approach for one-tap responses [76] and portable devices [94] given that many older adults may not always carry mobile phones or use stationary devices like Alexa for self-tracking [17]; and (3) *post-activity labeling*: supporting retrospective annotation by allowing users to backfill activities, as well as review and modify system-generated labels through features for deleting, editing, or adding labels. By distributing labeling efforts across multiple touchpoints, such a system could be integrated as a natural, low-friction part of everyday life, rather than disrupting daily routines, echoing accessibility goals outlined in prior work [79]. Moreover, a labeling system equipped with multimodal input could accommodate older adults with diverse abilities, enabling users to select the modality, such as touch, speech, or text, that best meets their accessibility needs and preferences [59, 65].

#### Consistent labeling.

Our participants perceived challenges in labeling with precision and consistency, particularly when distinguishing conceptually similar activities and applying labels consistently across different contexts. As activity settings and intentions shift, participants often need to reassess boundaries based on goals and interpretations of meaningful distinctions. Prior research by Yang et al. observed how participants monitor progress toward their activity goals through precision in activity tracking, defining precision as “consistency in measuring the phenomena of interest” [99]. Similarly, our participants aimed to ensure labeling consistency when training a model to reflect their activity goals. To support this need for consistent labeling, our findings suggest that intelligent activity labeling systems should (1) recognize contextual variations and prompt users when new labels may be needed; (2) visualize confidence levels for learned activities, helping users decide when to refine their labels; and (3) notify users when new labels do not align well with existing categories.

One promising technical approach is to employ a “consistency check” that flags potential labeling conflicts—for instance, when a new label might be redundant with or contradict an existing one. In such cases, large language models (LLMs) could detect semantic similarities between new and existing labels. If a user labels an activity as “pushing cart,” the LLM might identify its similarity to an existing “shopping” category and prompt for confirmation. LLM could also help reduce user burden by inferring or suggesting labels from users’ free-form descriptions. These features would promote better alignment between user-generated labels and the system’s model of activity classes. However, we argue that such prompting should remain aligned closely with users’ tracking goals, initiating only when it supports activities that users themselves consider important to track.

#### Flexible label granularity.

Another challenge our participants perceived was balancing the cognitive burden of labeling with the benefits of fine-grained data. Fine-grained, detailed labels may enhance model performance, but it requires users to recall or annotate highly specific details, which can be taxing—especially for complex or multi-step activities. Participants varied in how much detail they wanted to provide, often adjusting the level of specificity based on their tracking goals or perceived value of the data. Supporting this flexibility requires systems that help users understand the tradeoffs involved. For example, P2 expressed interest in understanding how the level of label detail would influence the system’s learning, highlighting a desire to engage more actively with the model training process. This aligns with prior work emphasizing the importance of communicating personal value and benefits before technology adoption among older adults [30, 58, 71, 74].

At the same time, more flexible labeling systems introduce tradeoffs. Providing too much freedom may reduce data consistency or label completeness, while rigid prompts for fine-grained labels may cause fatigue or disengagement. We suggest that systems incorporate adaptive prompting strategies that help users reflect on their labeling patterns and gradually adjust granularity over time—offering guidance without imposing strict requirements. Ultimately, we envision a bidirectional learning process in which both the user and the system adapt. For instance, users might begin by labeling complex activities like cooking, but later shift toward simpler, repeatable activities like dumbbell exercises that are easier to teach. The system, in turn, could learn which activities warrant detailed labeling and when to reduce granularity to support long-term engagement. Future work should support this dynamic process, enabling older adults to retain control over label specificity while allowing the system to adapt to their evolving needs and preferences.

### 5.2 Contributions to Human Activity Labeling Research

To clarify our contributions, we situate our work within the broader landscape of self-annotation approaches for training HAR models and articulate how our work advances this space, particularly through the lens of our older adult participants. We describe the distinctive aspects of our work across the target audience, context, and methodological design.

#### Target audience.

Our study foregrounds the perspectives of older adults, a population historically underrepresented in the development of activity labeling systems. Most existing activity labeling systems have been designed for and evaluated primarily with younger or middle-aged adults (e.g., [15, 19, 90, 91]), often assuming a baseline of comfort with mobile and wearable technologies. Given age-related differences in cognitive models and technology familiarity, it is important to conduct dedicated studies to understand how older adults approach activity labeling. Our work addresses this gap by engaging a group of older adults in an early design process to explore their labeling preferences. Our study shows that older adults in our participant group could meaningfully participate in machine teaching through data labeling, though misconceptions about how such systems learn can create barriers. For example, some participants assumed the machine would be capable of prompting them when an activity was omitted, not realizing it needed labeled training data to do so. This reflects broader challenges with AI literacy [63] and echoes prior work identifying inflated expectations of machine inference capabilities among non-expert users [38]. Addressing such misconceptions is essential for designing systems that not only support older adults but also help a wider range of users engage with AI technologies effectively and confidently.

#### Study context.

Our work is situated in the context of training a personalized activity recognition model through user-driven labeling—a framing aligned with machine teaching [101]. While a few instances have included older adults in machine teaching tasks [37, 60, 70], prior work centered on accessibility—involving blind older adults in the context of training object recognizers. Unlike image labeling, we focus on activity recognition, which requires mapping between time-series sensor data and corresponding activity labels. In this scenario, our participants retain control over which activities they label, as well as when and how they do so, enabling them to calibrate labeling effort against perceived benefits. This scenario contrasts with many prior HAR studies (e.g., [16, 47, 88]), in which participants were primarily positioned as data sources for researcher-driven goals, often without the ultimate goal of personalization or user agency. Our study shifts the framing toward end-user empowerment, exploring not only labeling mechanisms but also broader questions of motivation, interpretation, and trust.

#### Study design.

We adopt an exploratory, co-design approach, inviting participants to reflect on a range of labeling ideas, rather than evaluating a pre-defined system. This differs from prior studies that developed and deployed specific labeling tools (e.g., [19, 51, 90, 91]), where participant input is often constrained to post-hoc feedback. For instance, Cleland et al. used prompts based on motion state transitions, but did not examine participants’ preferences regarding timing or modality beforehand—leading to missed prompts [19]. Similarly, Kim et al. evaluated a speech-based labeling system with older adults, offering important insights into voice interaction but within a single-modality constraint [51]. In contrast, our study explored a broader range of labeling mechanisms, including input modalities, timing, review and error correction, and prompting strategies. Through the co-design process, participants provided insights into labeling behaviors, preferences, and constraints. The work most closely related to ours is by Tonkin et al., who investigated a multimodal approach to self-annotation but with younger and mid-aged adults [90]. Our work complements and extends this work by examining older adults’ perceptions and preferred strategies for collecting high-quality labels. Participants also articulated preferences for feedback features such as confidence scores and contextual information (e.g., time of day, posture, physiological signals), echoing prior work on “data descriptors” [37], which can support label interpretation and improve data quality. These insights suggest promising directions for building transparent, adaptive systems that make machine teaching more accessible and meaningful for older adults.

### 5.3 Older Adults’ Agency in Training Personalized Activity Trackers

Agency, often used with autonomy interchangeably, has served as an umbrella term for a sense of control and self-identity that ties to one’s values and goals in HCI [6]. In the context of self-tracking, agency is particularly important for older adults, as it enables them to actively manage their activity data, including how and with whom it is shared [7]. While prior research has highlighted the role of agency in promoting active and healthy lifestyles [31, 93], our work takes a more proactive approach by examining how older adults act as data collectors, directly shaping the underlying ML model of a personalized activity tracker.

Our findings build upon prior work examining customization in self-tracking [5, 34, 46, 52, 85] and envision mechanisms to support capturing accurate, precise, and granular activity labels, considering end users’ preferences. The customizing feature for data presentation and visualization supports users in integrating personal interests and self-identity in the self-tracking process [34, 46]. Similarly, instead of having a predefined set of universally used labels, future activity labeling tools should allow users to customize the labeling process (e.g., the label category) that caters to their activity goals and personal significance. Rather than relying on a predefined set of labels, we envision a personalized activity tracking system that allows users to define custom label categories aligned with their activity goals and personal significance. This approach parallels OmniTrack’s customization features, which enable users to create their custom trackers [52] and modify tracking fields as their goals evolve. Building on this flexibility, integrating a dynamic labeling regimen into older adults’ continuous labeling process could support their agency.

### 5.4 Reflecting on Participant Demographics and Study Limitations

We acknowledge that our participant demographics lean heavily toward highly educated individuals. Also, nine of our participants had prior experience participating in a research study that involved wearing a smartwatch device to report their activities. We invited these individuals to participate in our study because they met the inclusion criteria and expressed interest in participating in a future research study. We believe that their familiarity with the wearables likely enabled them to provide ecologically grounded insights, as they had first-hand experience of using wearables for activity labeling. However, their prior exposure to research and technology may also mean that our participants are not fully representative of the broader older adult population. We recruited the other three participants from an email list of a senior community in a different city. Although the majority of this group was generally comfortable using smartwatches, one participant found it difficult to envision interacting with multiple devices (e.g., smartphones, smart speakers) due to limited experience. We aimed to recruit a diverse group of participants in terms of technology proficiency and health conditions, and therefore did not apply exclusion criteria based on cognitive, sensory, or motor impairments. As reported in Section 3.2, our participants disclosed various health conditions, but none reported severe cognitive impairments. We acknowledge that older adults with cognitive impairment may have distinct needs, constraints, and strategies for activity labeling [87]. As such, we believe it is important for future work to meaningfully engage this population. For example, given the different contexts of daily living for older adults with cognitive impairments [61], we raise several questions for future investigation: What personalized activity recognition needs exist for this group? How feasible is it for them to collect high-quality activity labels? What labeling strategies do they prefer or require? And if self-labeling is not feasible, what roles might caregivers or care partners play in facilitating this process? We believe these questions merit dedicated and sustained research efforts.

Each co-design session was conducted with a single participant and two researchers, one with an HCI background and the other with ML. We chose to co-design with a single participant to encourage personalized solutions tailored to individual activity contexts rather than aiming for a one-size-fits-all recommendation. To foster an equal design partnership, we provided design probes in a semi-structured format. However, we recognize the limitations of introducing input modalities (i.e., voice input, text entry, gestures, list selection, responding to prompts) and device types (i.e., smartwatches, smart speakers, and mobile phones), derived from researchers’ initial brainstorming. For example, Moore et al. noted various features older adults dislike about wearable devices, such as frequent charging and battery life, discomfort caused by rigid bands and wearing in bed [69]. Future work should include various device forms (e.g., band-type devices or clip-ons) and investigate their pros and cons regarding wearability and maintenance. These options were left open for participants to discuss and challenge. In addition, we observed that our participants were generally motivated to provide high-quality labels because of their willingness to contribute to research and accepted our study goal to train a well-performing activity recognition model. Although we encouraged participants to consider the benefits of future activity trackers, the extent to which our study goal aligned with their intrinsic motivations remains uncertain. However, their active engagement and willingness to contribute suggest a strong interest in shaping the future of activity tracking technologies.

## 6 CONCLUSION

In this work, we investigated older adults’ perception of providing high-quality activity labels consisting of activity names and time spans. We conducted a co-design study with 12 participants (aged 64 to 93) by adopting a scenario-based design approach. We provided contextualized definitions of accuracy, precision, and granularity, which served as a guiding framework for analyzing participants’ perceptions of activity labeling practices. Our findings revealed that contextual differences and personal significance (e. g., goals and intentions) influence the accuracy and precision with which participants label their activity names. To further alleviate the labeling burden and enhance label quality, participants suggested that machines initiate prompts to ensure and verify completeness, suggest new activities for labeling, and probe for fine-grained activity semantics and contexts. Meanwhile, participants desired to maintain control by adjusting label granularity and customizing prompt timing. Understanding participants’ perceptions and preferred strategies for collecting high-quality labels can inform the design of future activity labeling systems that older adults can effectively and comfortably use.

## Figures and Tables

**Table 1.** Participants’ demographics and self-rated confidence in tech use.

ID	Age (Gender)	Latest occupation	Education	Self-rated technology confidence
P1	64 (M)	Senior manager	Bachelor’s	Very confident
P2	72 (M)	Landlord	Bachelor’s	Enjoy the challenge
P3	93 (F)	Piano teacher	Bachelor’s	Enjoy the challenge
P4	65 (F)	Human resources specialist	Bachelor’s	Very confident
P5	77 (F)	Rehabilitation counselor	Master’s	Very apprehensive
P6	83 (F)	Disability consultant	Master’s	A little apprehensive
P7	82 (F)	Policy analyst	Master’s	Very confident
P8	68 (F)	Administrator	High school degree	Very confident
P9	69 (F)	Technical training manager	Bachelor’s	Enjoy the challenge
P10	66 (M)	Regulatory specialist	Master’s	Enjoy the challenge
P11	65 (M)	Healthcare manager	Master’s	Very confident
P12	72 (M)	Lawyer	Ph.D./M.D.	A little apprehensive

**Table 2.** The general and contextualized definitions of accuracy, precision, and granularity in activity data labeling.

	General definition	Contextualized definition (for Activity name & Time span)
**Accuracy**	The closeness of a measured value to its true value	**Name:** Correctly identifying and labeling an activity as it truly occurs **Time span:** Recording the start and end times that closely align with the actual moments the activity began and ended
**Precision**	The consistency of repeated measurements	**Name:** The consistency with which similar activities (occurring under the same conditions) are labeled the same **Time span:** The consistent recording of an activity’s start and end times under the same conditions
**Granularity**	The level of detail or depth of the data	**Name:** The level of detail or depth in a label, whether referring to the semantics of an activity or its contextual specifics **Time span:** The finest unit of time captured (e.g., second, minute, hour)

**Table 3.** A summary of the findings: participants’ perceived challenges to achieve high-quality activity labels and participants’ preferred labeling and prompting strategies.

	Labeling Perception & Challenges	Labeling & Prompting Strategies
**Activity Name**	**Accuracy**: Universally agreed-upon accurate labels are difficult to achieve due to individuals’ perceptions and contextual differences in activities. **Precision**: The labeling process of clearly and consistently delineating boundaries between activities is shaped by personal significance (e.g., goals and intentions). **Granularity**: Labeling an activity at fine-grained is often associated with an increased user burden and uncertain benefits.	Ensuring label completeness through machine-initiated suggestions Identifying and prompting for new activities beyond existing labels Adjusting label granularity to manage the labeling burden Probing fine-grained contextual information from users
**Activity Time Span**	**Accuracy**: Demarcating activities is an inherent challenge for labeling due to the blurring boundaries between activities. **Precision**: The tolerance for deviations can vary among individuals based on their expectations for labeling accuracy. **Granularity**: Recalling fine-grained activity time spans demands significant memory efforts	Supporting immediacy and proximity while alleviating prompting intrusiveness Improving label completeness through prompting half-baked labeling Revealing the level of alignment between time span and machine-sensed data Recalling activity time span from names at different levels of granularity

## CCS Concepts:

• Human-centered computing → Empirical studies in HCI.
